# Fragile X Associated Primary Ovarian Insufficiency (FXPOI): Case Report and Literature Review

**DOI:** 10.3389/fgene.2018.00529

**Published:** 2018-11-27

**Authors:** Dorothy A. Fink, Lawrence M. Nelson, Reed Pyeritz, Josh Johnson, Stephanie L. Sherman, Yoram Cohen, Shai E. Elizur

**Affiliations:** ^1^Hospital for Special Surgery, New York, NY, United States; ^2^Mary Elizabeth Conover Foundation, Inc., McLean, VA, United States; ^3^Perelman School of Medicine, University of Pennsylvania, Philadelphia, PA, United States; ^4^University of Colorado, Denver, CO, United States; ^5^Emory University, Atlanta, GA, United States; ^6^Sheba Medical Center, Tel Hashomer and Tel Aviv University, Tel Aviv, Israel

**Keywords:** FXPOI, POI, fragile X syndrome (FXS), primary ovarian insufficiency, fertility, irregular periods

## Abstract

Abnormalities in the X-linked *FMR1* gene are associated with a constellation of disorders, which have broad and profound implications for the person first diagnosed, and extended family members of all ages. The rare and pleiotropic nature of the associated disorders, both common and not, place great burdens on (1) the affected families, (2) their care providers and clinicians, and (3) investigators striving to conduct research on the conditions. Fragile X syndrome, occurring more severely in males, is the leading genetic cause of intellectual disability. Fragile X associated tremor and ataxia syndrome (FXTAS) is a neurodegenerative disorder seen more often in older men. Fragile X associated primary ovarian insufficiency (FXPOI) is a chronic disorder characterized by oligo/amenorrhea and hypergonadotropic hypogonadism before age 40 years. There may be significant morbidity due to: (1) depression and anxiety related to the loss of reproductive hormones and infertility; (2) reduced bone mineral density; and (3) increased risk of cardiovascular disease related to estrogen deficiency. Here we report the case of a young woman who never established regular menses and yet experienced a 5-year diagnostic odyssey before establishing a diagnosis of FXPOI despite a known family history of fragile X syndrome and early menopause. Also, despite having clearly documented FXPOI the woman conceived spontaneously and delivered two healthy children. We review the pathophysiology and management of FXPOI. As a rare disease, the diagnosis of FXPOI presents special challenges. Connecting patients and community health providers with investigators who have the requisite knowledge and expertise about the *FMR1* gene and FXPOI would facilitate both patient care and research. There is a need for an international natural history study on FXPOI. The effort should be coordinated by a global virtual center, which takes full advantage of mobile device communication systems.

## Introduction

Primary ovarian insufficiency (POI) is part of the differential diagnosis for any woman of reproductive age who presents with irregular menstrual cycles or infertility. In women under the age of 40 years, POI is characterized by at least 4 months of unpredictable or absent menstrual periods and two serum follicle stimulating hormone (FSH) levels in the menopausal range at least 1 month apart (based on the particular laboratory reference range) (Nelson, 2009). In this case presentation, we describe a 20-year-old woman with fragile X associated primary ovarian insufficiency (FXPOI) who experienced a diagnostic odyssey of nearly 5 years despite: (1) genetic testing at an early age revealing a positive *FMR1* premutation carrier status; (2) ongoing oligo-amenorrhea; (3) elevated FSH levels; and (4) a family history of POI. The result was a 5-year delay in starting appropriate hormone replacement therapy. She was also treated with thyroid and psychiatric medications that may have been avoided with an appropriate diagnosis and hormone replacement regimen. Despite the diagnosis of FXPOI, the young woman conceived two healthy pregnancies without medical intervention while on hormone replacement therapy. Many women with POI and their clinicians do not realize that it is possible to conceive without medical intervention and do not understand the need for appropriate hormone replacement (Hipp et al., 2016).

Progress in rare disease research presents special challenges due to small, geographically dispersed patient populations and underlying clinical heterogeneity. Evidence supports a need to move beyond methodological methods to address these challenges and to begin to understand the patient perspective at a deeper level in order to develop more pragmatic approaches (Tingley et al., 2018). The traditional approach fashions a clinical case history, which becomes progressively abstracted from the patient’s experience and the context of its original telling. The patient becomes increasingly incidental and takes on what might be best described as an anonymous shadow in the course of events. This prevents a full appreciation of the patient narrative sense, which is fundamental to the care, clinical management of individuals over time, as well as to effective clinical research (Greenhalgh and Meadows, 1999). Therefore, we include experiential quotes from the patient in our case report.

## Case Report

In May of 2006 a 20 year-old woman presented to the National Institutes of Health (NIH) Clinical Center for evaluation. Her chief complaint was “I am not feeling like myself.” She reported experiencing hot flashes, night sweats, insomnia, occasional crying episodes, sadness, and an unpleasant jittery feeling. She had experienced loss of interest in activities she normally enjoyed. She also complained of waking up in the middle of the night with intense hunger. At age 18, she developed symptoms of severe depression that required her to take medical leave from her freshman year of college. Since then she was on numerous psychotropic medications and at the time of admission was on an extensive and complex regimen. By report of the patient and her mother, her depression had been relentless and difficult to treat.

Here is how the patient described the situation:

“I left my university on medical leave and spent my freshman year in bed or at doctors’ offices. No one knew what was wrong with me, so they kept referring me to different doctors and prescribing more medicines to treat the symptoms. The psych docs sent me to the medical docs and the medical docs sent me to the psych! It was the most frustrating, upsetting, and debilitating year of my life.”

Cascade genetic testing at 4 years old had uncovered the patient carried an *FMR1* premutation (100–110 CGG repeats). Her older brother was found to have fragile X syndrome by genetic testing at age 9 years. Her mother and aunt also carried an *FMR1* premutation and both had experienced “premature menopause.” The patient reported menarche occurred at age 11. She never established regular menses. She began taking the oral contraceptive at age 13 due to debilitating dysmenorrhea and menorrhagia. She stopped the oral contraceptives at age 16. From age 16 to 18 she experienced only “spotting” every 3 to 4 months. Between age 17 and 18 she began having night sweats. Her endocrinologist began her on levothyroxine replacement despite normal free T4 and TSH levels. Subsequent endocrinologic evaluation at a referral center suggested possible Cushing’s syndrome. She had an elevated morning serum cortisol, an elevated urinary free cortisol (twice the upper limit of normal), and an abnormal overnight dexamethasone suppression test.

On admission to the NIH Clinical Center, the woman had normal vital signs. Her body mass index (BMI) was 28.3 kg/m^2^. Physical exam revealed no stigmata of Cushing’s syndrome. Initial psychiatric consultation at the NIH concluded the young woman had a mood disorder due to her general medical condition, possibly primary ovarian insufficiency. Diurnal serum cortisol levels and 24-h urine free cortisol were normal. Transvaginal ultrasound findings were consistent with a diagnosis of POI [a 2 mm endometrial stripe, a very small left ovary (1.2 × 1.6 × 1.0 cm) with no visible follicles, and the right ovary could not be convincingly demonstrated]. Serum gonadotropins were in the menopausal range (FSH 46 and LH 26 IU/L). Serum estradiol was also in the menopausal range (23.3 pg/ml). Serum 21-hydroxylase antibodies, thyroid peroxidase antibodies, and thyroglobulin antibodies were all negative. Serum prolactin and MRI of the pituitary were normal. TSH and free T4 were normal.

The NIH discharged the woman with the newly recognized diagnosis of FXPOI. She began taking hormone replacement therapy (a daily dose of 100 microgram of estradiol by transdermal patch and cyclic medroxyprogesterone acetate by mouth 10 mg per day for the first 12 days of each month). She kept a menstrual calendar as instructed. After discharge her psychotropic medications and thyroid replacement were tapered, she menstruated regularly on the hormone replacement regimen, and her symptoms of anxiety and depression resolved.

Here is how the patient described the situation:

“Therefore, when NIH diagnosed me with FXPOI, I was thrilled. It was the first time in 3 years somebody understood me and could help me. All I wanted to do was to go to college and experience a typical 20-year-old life. I did, and it was fabulous!”

The young woman married in August 2012. She conceived shortly thereafter without medical intervention while on hormone replacement therapy. She terminated the pregnancy as prenatal genetic testing showed a full mutation in *FMR1* in the fetus. Thereafter, she conceived two more pregnancies without medical intervention while on hormone replacement therapy, one in 2013 and one in 2016. Shortly prior to the conception in 2016 a physician at an IVF clinic suggested she proceed to egg donation and quoted a current chance of conception of less than 0.1%. This is despite her history of having had two prior pregnancies subsequent to the diagnosis of FXPOI. Both pregnancies and deliveries were unremarkable with the birth of two healthy girls.

## Epidemiology and Genetic Counseling

The *FMR1* premutation is now an established cause of POI (Sherman, 2000). A premutation is defined as having 55–199 expanded CGG repeats located in the 5′ untranslated region (UTR) of the X-linked gene, *FMR1*. The frequency of women who carry a premutation is about 1/300 (Hunter et al., 2014). The diagnostic criteria required to confirm a diagnosis of POI are summarized in Supplementary Table 1. The indicated clinical assessments recommended after making the diagnosis are summarized in Supplementary Table 2.

The most immediate and significant consequence of FXPOI is reduced fertility (Allen et al., 2007; Streuli et al., 2009). POI occurs in about 20% of women with a premutation, making the risk of POI in this population about 20 times higher than the general population (Sherman, 2000; De Caro et al., 2008; Sullivan et al., 2011). Taking all women who carry a premutation, on average, they go through menopause about 5 years earlier than those without a premutation (Murray, 2000; Sullivan et al., 2005; Seltzer et al., 2012). Also, among women attending a reproductive endocrine clinic, about 11% with familial POI and about 3.2% with isolated POI are found to carry a premutation; thus, FXPOI is the most common genetic form of POI (Sherman et al., 2007).

There are at least three risk factors associated with the onset of FXPOI. The first is repeat size. Among premutation carriers, there is a non-linear relationship between CGG repeat length and risk for POI (Sullivan et al., 2005; Ennis et al., 2006; Allen et al., 2007; Tejada et al., 2008; Spath et al., 2011), irregular menses (Allen et al., 2007) and subfertility (Allen et al., 2007). For example, respectively, the risk for FXPOI is about 10, 32, and 16% for women with 55–79 repeats, 80–100 repeats, and greater than 100 repeats (Allen et al., 2007). The onset of POI is earliest among those with 80–100 repeats, sometimes as early as the adolescent years (De Caro et al., 2008). The risk for FXPOI is increased for women who report a family history of early menopause (Hunter et al., 2008; Spath et al., 2011), indicating background genetic variants as the second risk factor. Third, a woman who has ever smoked in her lifetime is subject to reduced age at menopause, although this effect among women with and without a premutation is similar (Allen et al., 2007).

Genetic counseling is essential once a woman is found to carry a premutation. In addition to the risk of FXPOI and its clinical consequences, each woman has a risk for having a child with fragile X syndrome. The magnitude of this risk is related to the number of CGG repeats identified in her FMR1 gene. The larger the number of repeats, the higher the risk for expansion from a premutation to a full mutation. There is also risk for developing another established premutation-associated disorder, fragile X-associated tremor/ataxia syndrome (FXTAS). FXTAS is a neurodegenerative disorder with an onset around age 60 (Hagerman et al., 2001). Although men who carry a premutation are more frequently affected by FXTAS, women have a lifetime prevalence of 6–18% (Wheeler et al., 2014).

### Diagnosis of POI

The median age of menopause in the United States is ∼51 ± 1 years, with 1% of women experiencing menopause prematurely (referred to as primary ovarian insufficiency, POI) (Palacios et al., 2010). POI is diagnosed when a woman has (1) experienced at least 4 months of unpredictable or absent menstrual periods before age 40 and (2) has two serum FSH levels in the menopausal range (Nelson, 2009).

### Diagnosis of a FMR1 Premutation

The overall methods to detect the expanded CGG repeat in the 5′ UTR of the FMR1 gene have not changed significantly over the years – all involve conducting PCR using probes that surround the repeat, followed by Southern blot analysis if required (Fu et al., 1991; Yu et al., 1991; Erster et al., 1992; Brown et al., 1993). Advances of PCR technologies have led to the ability to capture large premutation and full mutation repeats with accuracy, sometimes eliminating the need to follow the results with Southern blot analyses [e.g., triplet repeat-primed PCR (Tassone et al., 2008; Chen et al., 2010; Filipovic-Sadic et al., 2010; Hantash et al., 2011)]. All diagnostic methods are reviewed in Standards and Guidelines for Clinical Genetics Laboratories of the American College of Medical Genetics and Genomics, which were most recently updated by Monaghan et al. (2013).

The most recent advance in characterizing the FMR1 repeat expansion is inclusion of the AGG interruption pattern within the CGG repeat (reviewed in Latham et al., 2014). The finding that the CGG repeat was interrupted with periodic single AGG sequences in the 5′ region of the FMR1 repeat was first described by Eichler et al. (1994). Once an accurate PCR-based method to deduce the AGG interruption pattern was developed in 2010 (Chen et al., 2010), several large studies were conducted and showed clearly that the repeat structure, including repeat length and AGG interruption pattern, altered the risk for instability of the repeat during transmission from parent to child (Yrigollen et al., 2012, 2013; Nolin et al., 2013, 2015). Whether or not this added information about repeat structure alters the risk for FXPOI is not clear, as studies have shown conflicting results (Lekovich et al., 2017; Allen et al., 2018).

## Basic Science

**FIGURE 1:**
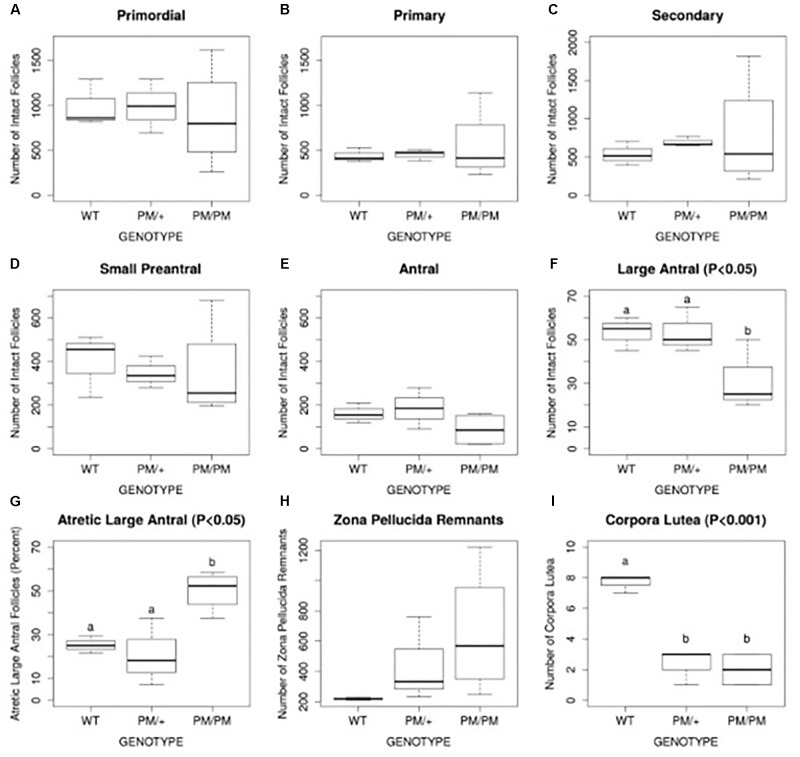
Histomorphometric analysis of follicle number, atresia, and corpus luteum number of 8 month old FXPM mutants and wild type control animals. Quantification of intact primordial, primary, secondary, pre-antral, and large antral/periovulatory follicles per ovary (*n* = 4 per genotype) is shown in **A–F**. Follicle atresia was evaluated by counting atretic large antral follicles **(G)** and zona pellucida remnants (ZPR, **H**). Corpus luteum (CL) number is shown in **(I)**. Note increased variability in the number of PM/PM follicles, ZPR, and CL. Statistically different means, where applicable, are denoted by letters “a” and “b,” with *P*-values shown above each plot as calculated by ANOVA analysis. Conca Dioguardi et al. (2016).

Evidence suggests the most common mechanism of FXPOI is one of abnormal follicle function rather than a depletion of primordial follicles. There is limited histologic evidence regarding the ovarian pathology in women with FXPOI. One small study showed no significance difference from normal control ovaries with regard to ovarian histology or follicle number (Chang et al., 2011). These data are consistent with the mechanism of FXPOI being mainly one of follicle dysfunction rather than follicle depletion. These human findings are consistent with histologic evidence in mouse models of FXPOI. As shown in Figure 1, mice carrying an *FMR1* premutation had a normal number of primordial follicles. The significant differences from control mice were: (1) fewer large antral follicles, (2) fewer corpora lutea, and (3) a greater number of atretic large antral follicles. (Figure 1; Conca Dioguardi et al., 2016). Thus, available evidence suggests FXPOI is a primarily a disorder of impaired follicle function.

Published data are available about FXPOI models in three separate mouse strains (Sherman et al., 2014). The first, the “NIH” mouse, harbors 130 CGG repeats (here, “130R”) in the Fmr1 5′ UTR (Entezam et al., 2007). The second, referred to here as the “90R” mouse, has 90 repeats (Peier and Nelson, 2002). Newer data from the third “Dutch” CGG knock-in strain are also discussed.

Reproductive and ovarian parameters have largely been consistent between the 130 and 90 R strains (Pastore and Johnson, 2014). This includes negative impacts upon ovarian follicle growth and survival that correspond to decreased litter size(Lu et al., 2012; Conca Dioguardi et al., 2016). Further, alterations in the levels of hormones estradiol and FSH have been detected in the 90 R mouse (Lu et al., 2012). Additionally, the growth of ovarian follicles is slower due to an elevation in the follicular apoptotic index (Uslu et al., 2017). Ultimately, the number of cumulus granulosa cells in mature follicles, and, those ovulated with the egg from mature ovulatory follicles, are decreased in number (Hoffman et al., 2012). Both models are also associated with increased follicle atresia (Hoffman et al., 2012; Lu et al., 2012).

All of the above features of compromised growth and function of mouse follicles were recently found to correspond to reduced mitochondrial (mt) DNA copy number, total mitochondrial mass, and altered expression of genes that control mt function (Conca Dioguardi et al., 2016). Causal links between the premutation, sub-functional mt, and follicle and ovary function remain to be elucidated. Interestingly, mt abnormalities have also been detected in other tissues (Alvarez-Mora et al., 2016; Giulivi et al., 2016), including those from women who carry a premutation.

In a separate study using ovaries from the “Dutch” exCGG-KI mice (100–199 CGGs), Buijsen et al. (2016) found evidence for translation of the CGG-bearing Fmr1 RNA, resulting in the production of Repeat-associated non-AUG (RAN)-translated poly-glycine species (Kearse and Todd, 2014). These aberrant translation products have been detected in neuronal intracellular inclusions that correspond to the pathology seen in FXTAS (Sellier et al., 2017). Buijsen et al. (2016) detected RAN inclusions within ovarian stromal cells in the “Dutch” mouse, as well as the ovarian stromal of a woman with FXPOI. Taken together, this suggests that RAN translation could contribute to ovarian pathophysiology as well.

## Endocrinology

The ovary develops a graafian follicle from a primordial follicle every month. After ovulation, a corpus luteum forms (Hawkins and Matzuk, 2008). These secretory structures produce a complex symphony of reproductive hormones, which create the menstrual cycle. After the diagnosis of POI, about 5–10% of women have a spontaneous conception (van Kasteren and Schoemaker, 1999; Nelson et al., 2005). A more recent study noted a spontaneous conception percent of 12.6 specifically in women with FXPOI (Hipp et al., 2016).

The goal in treating women with POI is to optimize their health in an integrated manner and to replace their missing reproductive hormones in the most physiologic manner possible (Sullivan et al., 2016). In women of reproductive age who are having regular menstrual cycles the average serum estradiol level across the menstrual cycle is about 100 pg/mL (Mishell et al., 1971). The 100 mcg per day estradiol patch and vaginal ring deliver an appropriate amount of estradiol to maintain this serum level. Not only does this approach normalize serum estradiol levels, but also the regimen frequently normalized serum LH levels. A study of 137 women with spontaneous POI demonstrated that the transdermal estrogen patch at a dose of 100 mcg/day normalized LH levels in approximately half of the women (Popat et al., 2008). Normalizing serum LH levels is an important consideration because the most common mechanism of follicle dysfunction in women with POI is inappropriate follicle luteinization (Popat et al., 2008; Nelson, 2009). Theoretically, normalizing serum LH levels would improve the chance of ovulation.

**FIGURE 2:**
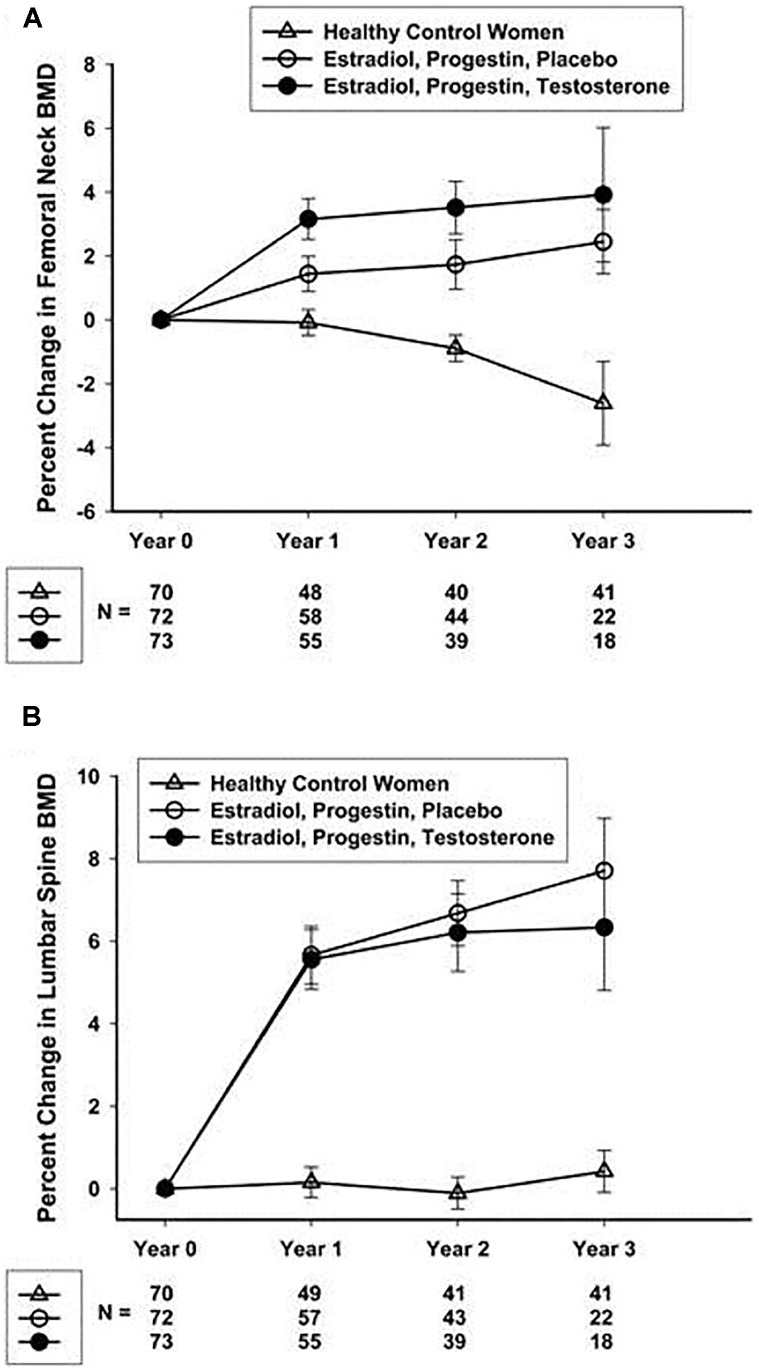
Percentage change over 3 years in femoral neck **(A)** and lumbar spine **(B)** BMD in healthy control women and women with 46,XX sPOI treated with E + P or E + P + T. Popat et al. (2014).

Studies focusing on POI and bone health have helped to define an optimal hormone replacement regimen. Fifty-nine women with spontaneous POI participated in a 2-year open randomized trial comparing physiologic hormone replacement therapy to oral contraceptive pills. The study found that women taking physiological hormone replacement therapy had significantly increased lumbar spine bone mineral density compared to women taking the oral contraceptive (Cartwright et al., 2016). Oral contraceptive pills are not as effective as physiologic hormone replacement therapy at improving or maintaining bone density. An NIH 3-year randomized controlled trial in women with spontaneous 46,XX (normal karyotype) POI demonstrated a 7.7% gain in femoral neck BMD with physiological transdermal estrogen and oral medroxyprogesterone replacement (Popat et al., 2014; Figure 2). The women in this study receiving the transdermal estrogen and oral medroxyprogesterone treatment had a mean age of 33 years. This study is striking because in normal women with regular menses, peak bone density is not reached until the early 30 s. The NIH study is good news for young women with POI. Even when women with POI develop an estrogen deficient state during the time of peak bone mass accrual, they can still regain lost BMD to normal over 3 years of replacement. On average, at the end of the NIH study period the BMD of women with POI did not differ from the control women with regular menses. Of note, a study evaluating hormone replacement regimens in ovariectomized rats showed the best outcome for vertebral BMD was when estrogen and progesterone were administered sequentially rather than in a continuous/combined approach as in oral contraceptive pills (Vanin et al., 1995). This is possibly the reason cyclic physiologic hormone replacement has a better effect on bone than oral contraceptive pills in women with POI.

**FIGURE 3:**
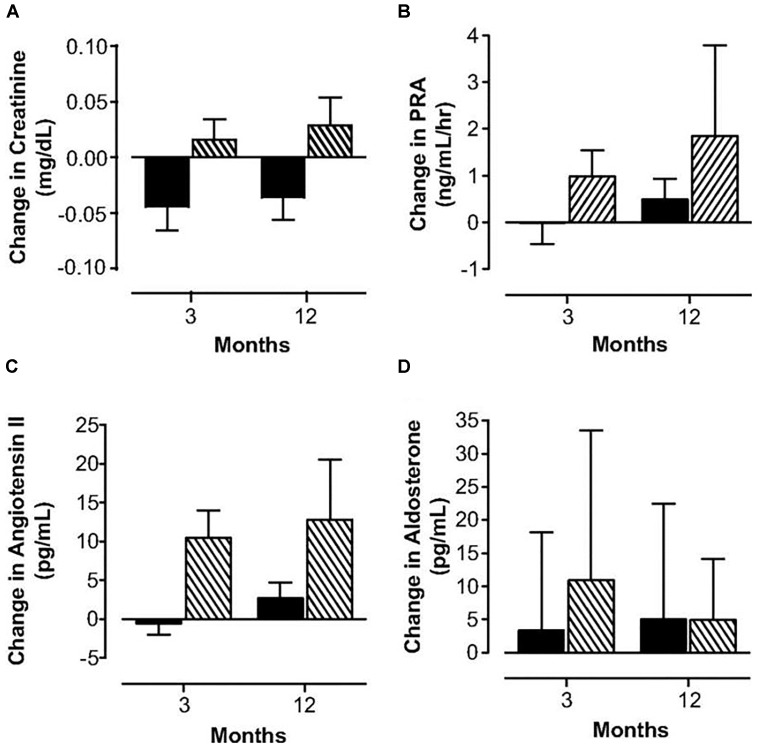
Changes in serum creatinine **(A)**, plasma renin activity **(B)**, angiotensin II **(C)** and aldosterone **(D)** concentrations in women with POI treated with physiologic HRT (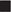) or standard OCPs (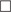). Langrish et al. (2009).

Oral contraceptives provide supraphysiologic doses of hormones and provide a continuous dose of estrogen and progestin, typically for 3 weeks followed by one week of placebo. They also induce unwanted physiologic changes as compared to physiologic hormone replacement (Langrish et al., 2009; Figure 3). Many women do not realize that oral contraceptive pills provide higher effective doses of estrogen and progestin compared to physiologic hormone replacement of estrogen and progestin. For women with POI who desire pregnancy, oral contraceptive pills are a problem as they induce hostile cervical mucus (Steward et al., 2012) as well as atrophic endometrium (ESHRE Capri Workshop Group, 2001).

The normal ovary also produces testosterone (T). Free T levels are reduced in women with POI (Kalantaridou et al., 2006). In another domain, a meta-analysis of 42 studies demonstrated normal women who take oral contraceptive pills have reduced circulating levels of total T and free T as well as an increase in SBHG concentrations (Zimmerman et al., 2014). The use of oral contraceptives as hormone replacement therapy would thus be expected to further reduce free T levels, mediated by increased levels of SHBG.

The North American Menopause Society 2017 hormone therapy position statement recommends hormone therapy until the median age of menopause, which is 52-years-old in the United States (The NAMS 2017 Hormone Therapy Position Statement Advisory Panel, 2017). Unfortunately, hormone treatment is often delayed or inappropriately administered to women with POI. A study of 79 women with FXPOI found that (1) many women took hormones for less than a year or never received hormone replacement, (2) had a greater than 1 year delay in beginning hormone replacement after the POI diagnosis, or (3) discontinued hormone replacement therapy before age 45 years (Hipp et al., 2016).

## Behavioral Health

Emotionally, POI can be a devastating diagnosis for women. Compared to controls women with POI score adversely on measures of anxiety, depression, as well as positive and negative affect. In controlled study, measures of illness uncertainty and purpose in life were significant independent factors associated with anxiety. Also, measures of stigma and purpose in life were significant independent factors associated with depression. Further, measures of goal reengagement and purpose in life were significant independent factors associated with positive affect. These findings suggest clinicians could help women with POI improve their quality of life by: (a) informing them better about their diagnosis, (b) helping them feel less stigmatized by the disorder, and (c) assisting them in developing alternative goals with regard to family planning as well as other life goals. In a study evaluating depression in women with POI, women with idiopathic POI had a much higher incidence of depression than women with Turner Syndrome who had POI (Schmidt et al., 2011). In a separate study, many women with idiopathic POI had depression that began during the time of menstrual cycle irregularity, which preceded the diagnosis of POI (Schmidt et al., 2011). In addition, the patient–physician interaction sometimes causes significant emotional distress. A study evaluating the emotional needs of women at the time of POI diagnosis found that most of them were not satisfied with how their physician informed them about the diagnosis (Groff et al., 2005). As a result of the emotional stressors that occur at the time of diagnosis with POI there is a need to address these issues in an integrated manner (Covington et al., 2011).

## Reproduction and Fertility Preservation

Fragile X syndrome (FXS; OMIM 300624) is a common form of X-linked intellectual and developmental disability (Crawford et al., 2001) with a prevalence of 1/4000 – 5000 in males and 1/6000 – 8000 in females (de Vries et al., 1997; Crawford et al., 2001; Coffee et al., 2009; Hill et al., 2010). It belongs to a family of more than 40 disorders characterized by repeat instability on transmission from parent to child (Pearson et al., 2005).

Most cases of the syndrome result from expansion of a CGG trinucleotide repeat located in the 5′ UTR of the FMR1 gene to more than 200 repeats (Oberlé et al., 1991; Verkerk et al., 1991; Yu et al., 1991). *FMR1* alleles with this expanded repeat are referred to as the full mutation. In a response to the expanded repeat, the *FMR1* gene undergoes locus-specific hypermethylation and chromatin remodeling that epigenetically silences the gene. Many alleles in the premutation range (55–199 CGG repeats) are remarkably unstable and at risk for full mutation expansions even in one generation. As many as 94% of alleles with more than 90 repeats expand to a full mutation (Nolin et al., 2011). Expansion to a full mutation occurs almost solely in transmission from mother to child and not from father to daughter although rare exceptions have occurred (Zeesman et al., 2004).

For this reason, in some countries, women who wish to avoid the risk of having a child affected with FXS are offered preconception genetic screening for FMR1 premutation. Currently, FMR1 premutation carriers who wish to conceive and avoid the risk of having an affected child have three options:

(1) To conceive using *In Vitro* Fertilization and preimplantation genetic testing for monogenic gene diseases (IVF-PGT-M).
(2) Spontaneous conception and prenatal genetic diagnosis during pregnancy by either chorionic villous sampling (CVS) or amniocentesis (AC).
(3) Using a donor oocyte.

Each approach has its advantages and disadvantages. Spontaneous conception carries a risk of bearing an affected child and the need to perform a termination of pregnancy. Termination of pregnancy involves medical, emotional and ethical issues. On the other hand, IVF using PGT-M avoids the need for a termination of pregnancy, and offers the opportunity to transfer only non-carrier embryos. However, this procedure arouses financial and emotional difficulties and is not the obvious choice for a fertile couple, especially considering the higher prevalence of ovarian dysfunction and reduced ovarian response observed in FMR1 premutation carriers undergoing IVF compared to non-carrier women (Elizur et al., 2014).

In addition to the risk of having a child affected with FXS women who carry an *FMR1* premutation may suffer from ongoing deterioration of ovarian function. This can be demonstrated by various markers such as high serum follicular phase FSH levels (Murray et al., 1999) low serum Inhibin A, Inhibin B (Welt et al., 2004), Anti-Mullerian hormone levels (AMH) (Spath et al., 2011) and low antral follicle count (AFC) (Elizur et al., 2014).

In the western world, there has been an overall increase of mean maternal age as a result of delayed childbearing (Mathews and Hamilton, 2016). Thus, it is essential to identify in a timely manner women who carry a premutation and are at risk for developing diminished ovarian function. This knowledge will offer these women the opportunity to make an informed decision regarding their reproductive and family planning. Some might pursue childbearing earlier than first planned or choose fertility preservation options.

**FIGURE 4:**
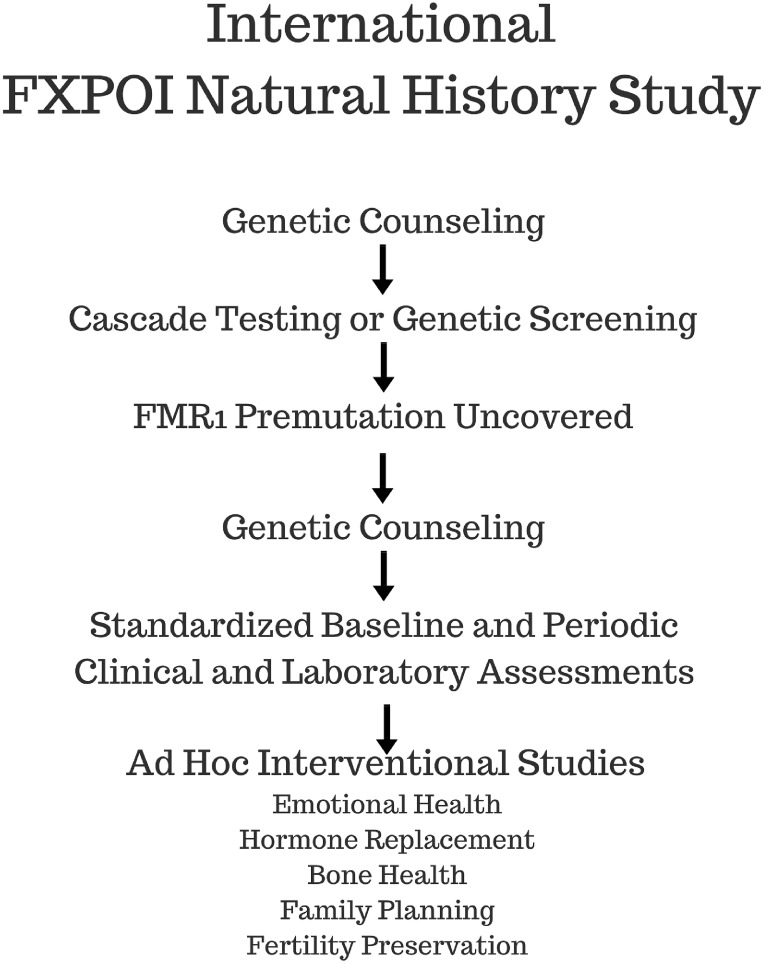
Schematic flow diagram depicts the process to be followed by the proposed International FXPOI Natural History Study.

There is a need for an International FXPOI Natural History Study (Figure 4). Unfortunately, today, we do not have the ability to prevent or reverse the impaired ovarian function associated with FXPOI. However, we can take advantage of the latest developments in the rapidly evolving field of fertility preservation. Emphasis should be on early identification of women with a premutation and diminished ovarian function at the primary care level. Embryo and oocyte cryopreservation are currently the best available fertility preservation option for women who carry a premutation. Ovarian tissue cryopreservation is used today mainly as fertility preservation option for young women facing gonadotoxic treatment due to malignancy. It requires two surgeries: one to harvest the ovarian tissue and the second to implant it back when the woman heals from her primary disease. It is most successful in young women with normal functioning ovaries who face an acute and isolated potential damage to the ovaries. It is uncertain at this time whether women who carry a premutation will benefit from such a procedure.

As mentioned previously, it should be noted that FXPOI should not be equated with menopause. Ovarian function can still be present in women with established FXPOI, albeit the function is intermittent and unpredictable. Hipp et al. (2016) reported that 12.6% of women diagnosed with FXPOI conceived spontaneously after diagnosis. The time to conception after diagnosis ranged up to 12 years.

The new site-specific genomic editing tool, the CRISPR (clustered regularly interspaced short palindromic repeats) system, has recently been developed and implemented to target and mutate specific genomic regions (Cong et al., 2013). Xie et al. (2016) utilized the CRISPR genome editing technology to excise the expanded CGG repeat from the full mutation allele in FXS cells resulting in an *FMR1* allele without CGG repeats. The excision of the expanded CGG-repeat from the fragile X chromosome resulted in FMR1 reactivation thereby restoring FMRP production. Liu et al. (2018) applied recently developed DNA methylation editing tools and demethylated the CGG expansion by dCas9-Tet1/single guide RNA (sgRNA). This switched the heterochromatin status of the upstream FMR1 promoter to an active chromatin state, restoring a persistent expression of FMR1 in FXS iPSCs. These new developments in gene editing technology may offer us in the future the option to “cure” in the laboratory FXS affected embryos of FMR1 premutation carriers undergoing IVF.

## Moving Forward

Engaging a collaborative team is the most efficient and productive manner in which to conduct research on genetics and clinical care. This is true for virtually all disorders, and especially those which are relatively rare and pleiotropic (Harris et al., 2016). One effective way to stimulate and organize research at all levels is to develop centers of excellence (Martin et al., 2017). The National Fragile X Foundation has established a Fragile X Clinical and Research Consortium [FXCRC] (2018). Such centers can attract patients and their families for specialized care and research regarding all of the organ systems that might be affected. The patient described in this report initially required the services of psychiatry, endocrinology, reproductive endocrinology, medical genetics and genetic counseling. Later in life, neurology might become involved related to FXTAS.

All of these evaluations and management plans would best be coordinated by a clinician who understands the underlying mutation and its multiple effects (Rafique et al., 2012). Centers of excellence, typically at academic medical centers, are well positioned to serve all of the clinical needs and potential therapies for those with fragile X-associated disorders. Through regular meetings of the center’s health care professionals, individual patients can be discussed and new clinical management issues planned. Often such discussions lead to stimulation of clinical studies, even clinical trials (Falorni et al., 2014). Moreover, individual members of a center often interact with basic science colleagues, engage them with ideas, and sometimes stimulate them to undertake pertinent research.

## Summary and Conclusion

Fragile X-associated primary ovarian insufficiency (FXPOI) is a chronic disorder characterized by oligo/amenorrhea and hypergonadotropic hypogonadism before age 40 years. There may be significant morbidity due to: (1) depression and anxiety related to the loss of reproductive hormones and infertility; (2) reduced bone mineral density; and (3) increased risk of cardiovascular disease related to estrogen deficiency. We report the case of a young woman who never established regular menses and yet experienced a 5-year diagnostic odyssey before establishing a diagnosis of FXPOI. This is despite a known family history of fragile X syndrome and early menopause in the family. Despite having clearly documented FXPOI, the woman spontaneously conceived and delivered two healthy children. This is consistent with the pathophysiology of FXPOI being primarily a situation of ovarian follicle dysfunction rather than ovarian follicle depletion. As a rare disease, the diagnosis of FXPOI presents special challenges. There is a need for increased awareness of this disorder among health care professions. International centers of excellence may be helpful to address the needs of families dealing with the sequelae of abnormalities in *FMR1*. Such centers should be coordinated by a global virtual center, which takes full advantage of mobile device communication systems.

Such an approach would put patients and community health providers in touch with investigators who have the requisite knowledge and expertise about the *FMR1* gene and its various manifestations. This would facilitate patient care and research on an international level. One of the top priorities of centers would be to conduct a natural history study on FXPOI.

## Author Contributions

DF organized the abstract, endocrinology, behavioral health, and conclusions section. LN organized the abstract, case report, and conclusions. RP contributed to the reproduction and fertility preservation section. JJ organized the basic science section. SS organized the epidemiology and genetics section. YC contributed to the reproduction and fertility preservation section. SE contributed to the reproduction and fertility preservation section.

## Conflict of Interest Statement

The authors declare that the research was conducted in the absence of any commercial or financial relationships that could be construed as a potential conflict of interest.
