# Supplementary material for: Fragile X Associated Primary Ovarian Insufficiency (FXPOI): Case Report and Literature Review
Source: Front Genet. 2018 Nov 27;9:529. doi: 10.3389/fgene.2018.00529 (PMC6278244; doi:10.3389/fgene.2018.00529)
Supplement: Supplementary file 1 [file Table_1.DOCX]

Table 1 FMR1 Manuscript Israel

**Table 1**

**Criteria to establish a diagnosis if Primary Ovarian Insufficiency (POI)**

- Age less than 40 years
- Oligo/amenorrhea for at least 4 months
- Two serum FSH levels
  - In the menopausal range as defined by the laboratory
  - Measured at least one month apart
