# Supplementary material for: Fragile X Associated Primary Ovarian Insufficiency (FXPOI): Case Report and Literature Review
Source: Front Genet. 2018 Nov 27;9:529. doi: 10.3389/fgene.2018.00529 (PMC6278244; doi:10.3389/fgene.2018.00529)
Supplement: Supplementary file 2 [file Table_2.DOCX]

Table 2 FMR1 Manuscript Israel

**Table 2**

**Clinical Evaluation of Confirmed Primary Ovarian Insufficiency (POI)**

- Karyotype (count 30 cells to detect mosaicism)
- Adrenal Antibodies
  - 21-hydroxylase (CYP21) by immunoprecipitation
  - Indirect immunofluorescence
- FMR1 Premutation
- Pelvic Ultrasound
- Bone Mineral Density
